# Hints in Operating for Fistula in Ano

**Published:** 1882-03

**Authors:** J. M. Mathews


### SELECTIONS.

### HINTS IN OPERATING FOR FISTULA IN ANO.

Very much of the failure to cure rectal disease, I imagine, is due to wrong impressions or ideas as to the cause and nature of the same. Authorities have differed upon the subject of the pathology of fistula in ano, even from the very first who wrote of the disease. Brodie taught that it always originated from ulceration of the gut, and that, therefore, there always existed an internal opening from the first. Syme taught that the mucous membrane was always intact at the beginning of the trouble, and only developed when suppuration existed. The fact is that both of these authorities were wrong. A foreign body may be the starting, or cause of a fistula, either from the inside of the rectum or from the outside. There can exist internal openings without any ulceration of the mucous membrane.

In giving any "hints" of the operation for this trouble, I, of course, mean to speak of patients who suffer from no special diathesis, or other trouble which might prove a complication, or obstacle in the way of the operation.

There are four modes of operating for fistula in ano:

1. Injecting the sinus.
2. The silk ligature.
3. The elastic ligature.
4. The knife.

I speak of the injection of the sinus as a *mode*, from the fact that it is yet practiced by the empirics to a great extent. This is done, no doubt, to save the feelings of the patient who may object to the use of the knife, caustic (?) or ligature. In operating upon a *recent* fistula where no induration of the tissues exist, I have no doubt but that some good can be done by employing injections of silver nitrate, iodine, etc., into the tract, especially if the condition be sluggish, and shows no disposition to heal. To say that the adoption of it, in fistulas of any length of standing, is correct treatment, is simply folly. Irritation sufficient to close up the external orifice, might be excited, but this, in nine cases out of ten, is just the thing you do not want to do. To effect this much is not *curing* the disease, and no surgeon would risk his reputation in this manner.

2. The use of the silk ligature is still in vogue, particularly among the older members of the profession. It has its advantages, and, in some cases, we can not afford to ignore its use. In cases where any special diathesis exists, say scrofulous, or tuberculous, and the use of the knife is contra-indicated, for fear of incontinence, hemorrhage, pain, etc., this can be said to be a very proper, if not satisfactory, mode of operation. Patients dread the use of the knife; this mode overcomes their objections, and is comparatively free from pain, another advantage being that they can be kept out of bed at their usual avocation. The method of applying it is of course understood, it being simply to thread a small flexible probe, pass it through the external opening into the bowel, thence out at the anus, and tie a little *firmly*, but not tightly. It is re-tied every few days, according to the circumstances. If only one sinus exists, a cure may be affected. If the disease has existed for any length of time or the tract be callous, the operation will be ineffectual.

3. The elastic ligature is highly lauded by some; but to say that it can be successfully used indiscriminately, and that to the exclusion of the knife, is saying something that can not be supported by facts. This mode of operating has its advantages, yet, it has its disadvantages. To state briefly, its advantanges are, viz: It does away with the horror of the knife; it is not attended by hemorrhage; it can be used *sometimes*, with chloroform; it causes less pain if not drawn *tightly*; and the patient can usually go about during the treatment. Its disadvantages are: If the sinus is callous, it will not cure the disease; if, according to all authorities, the ligature is drawn tightly, the pain is intense; it delays the cure very many days, from the fact that it takes from eight days to three weeks to cut out; if the disease has existed for any length of time, its use is contra-indicated.

4. The knife is undoubtedly to be preferred to all other methods, in the great majority of cases. No surgeon should succumb to the whims of his patient, if in so doing he violates his own judgment. Therefore, a patient should never be allowed to dictate an operation or how to perform it.

The great majority of cases of fistula in ano are not seen until after they have existed for some time. We all know that if this be true, that the great majority of them have *calloused* tracts. If you use injections, you only close up the external opening. If you use the ligature, either silk or elastic, you cut through the top and lose the bottom, the consequence being that you do no good—the sinus opening again in a little time. If you use the knife, you cut through not only the top but the *bottom*, which is your duty to do, and you cure your patient. If other sinuses exist beside the main one—if you use the knife, you lay them all open, and the patient gets well; if you use the ligature, you can not see the sinuses, and your patient does *not* get well. If you use the knife, and the edges of the wound need trimming, you can trim them; if you use the ligature, you can not do this. The old authorities used to talk about *cutting out* a fistula—the laity have such an impression yet. I tell you, gentlemen, there is more truth than poetry in the idea. The more you cut these patients the better they do, and if I could not get "shut" of a callous sinus any other way, I would *cut* it out. As to the employment of chloroform in operating for these troubles, I would say that I prefer to use it in most of the cases, for the reason not only that it relieves the patient of pain, but that it permits the operator to cut as much as he sees proper.—*Prof J. M. Mathews in the Louisville Med. Herald.*

